# Serum IL2Rα as a Diagnostic Biomarker and Its Functional Interplay With IL‐2 in Breast Cancer

**DOI:** 10.1155/tbj/5295439

**Published:** 2026-07-22

**Authors:** Wei Wang, Jun Liu, Yun Wei, Yan Fang, Shuning Ding, Deyue Liu, Li Zhu

**Affiliations:** ^1^ Department of General Surgery, Shanghai General Hospital, Shanghai Jiao Tong University School of Medicine, 100 Haining Road, Shanghai 200080, China, shsmu.edu.cn; ^2^ Department of Nursing, Shanghai General Hospital, Shanghai Jiao Tong University School of Medicine, 100 Haining Road, Shanghai 200080, China, shsmu.edu.cn

**Keywords:** biomarker, breast cancer, cytokines, IL2Rα, immune infiltration

## Abstract

**Background:**

Breast cancer is a leading female malignancy, with chronic inflammation driving progression. The IL‐2/IL‐2R axis regulates immunity, but IL2RA’s role in breast cancer (expression, diagnostic/prognostic value, and interplay with IL‐2) is unclear.

**Methods:**

A total of 252 consecutive patients (99 breast cancer and 153 benign breast disease) who received breast surgery were retrospectively analyzed. Serum cytokines were measured via electrochemiluminescence immunoassay; ROC, single‐cell sequencing, and bioinformatics databases (TIMER and Kaplan–Meier plotter) were used to assess IL2RA/IL‐2’s utility and associations.

**Results:**

Serum IL2Rα was higher in breast cancer (336.9 vs. 275.8 U/mL, *p* < 0.001; no significant differences were observed for other cytokines). IL2Rα showed moderate diagnostic performance (AUC = 0.650, cut‐off = 240.5 U/mL), correlated with NLR (*p* = 0.012), and showed a trend towards poor survival (*p* = 0.124, HR = 0.309, 95% CI: 0.069–1.381). Single‐cell sequencing identified T cells as the primary source of IL2Rα in tumor tissues. High IL2RA expression strongly correlated with infiltration of immune cells, especially neutrophils (*r* = 0.673) and dendritic cells (*r* = 0.706). RNA‐seq analysis showed IL2RA was upregulated in tumor tissues, associated with poor prognosis (*p* = 9.7*e* − 06, HR = 1.29, 95% CI: 1.15–1.44) and aggressive features (e.g., lymph node metastasis and HER2 positivity). In contrast, IL‐2 was highly expressed in normal tissues, linked to better prognosis (*p* = 0.001, HR = 0.83, 95% CI: 0.74–0.93), and had opposing clinical correlations.

**Conclusions:**

Serum IL2Rɑ could serve as a complementary auxiliary diagnostic biomarker for breast cancer, with associations with systemic inflammation, immune cell infiltration, and aggressive disease. The functional antagonism between IL2RA and IL‐2 highlights the IL‐2/IL2RA axis as a critical regulator of breast cancer pathophysiology, supporting its potential as a therapeutic target for improving breast cancer diagnosis and treatment.

## 1. Introduction

Breast cancer remains the most prevalent malignancy among women globally, posing a substantial threat to public health. As reported by the World Health Organization in 2022, over 2.26 million new cases are diagnosed annually, with approximately 685,000 deaths attributed to the disease [1]. While the etiology of breast cancer involves a complex interplay of genetic, environmental, and lifestyle factors, accumulating evidence highlights chronic inflammation as a critical driver of tumor initiation and progression [2, 3]. Inflammatory processes modulate tumor development through the secretion of cytokines, soluble proteins that regulate intercellular communication, and immune responses [4]. Cytokines of the interleukin (IL) family, including IL‐1β, IL‐6, and IL‐8, have been implicated in breast cancer pathogenesis, particularly in processes such as bone metastasis and tumor cell proliferation [5, 6].

Currently, clinically available noninvasive biomarkers for the auxiliary diagnosis of breast cancer remain limited. There is an urgent need to identify novel and reliable serum indicators to improve the early identification and risk assessment of breast cancer. Among the cytokines involved in immune regulation, the interleukin (IL‐2)/IL‐2 receptor (IL‐2R) axis plays a pivotal role in lymphocyte activation, proliferation, and immune homeostasis [7–9]. IL‐2Rα, the α‐subunit of the high‐affinity IL‐2R, is constitutively expressed on lymphoid neoplastic cells and transiently induced on activated normal lymphocytes, serving as a marker of immune activation [10, 11]. Previous studies have noted elevated soluble IL‐2R levels in familial breast cancer patients, suggesting a potential association with breast cancer pathogenesis [12]. However, the precise role of IL2RA in breast cancer—including its serum expression patterns, diagnostic and prognostic value, cellular sources within tumor tissues, and interactions with the tumor immune microenvironment—remains largely unclear.

Furthermore, the functional relationship between IL2RA and its ligand IL‐2 in breast cancer requires clarification. IL‐2 is a key regulator of T cell development, B cell activation, and natural killer cell function, and its role in tumor immunity appears context‐dependent [13]. While some studies have reported altered IL‐2 and soluble IL‐2R levels in breast cancer patients [12, 14], their coordinated effects on disease progression and patient outcomes remain poorly understood.

In this study, we aimed to systematically investigate the clinical and biological significance of IL2RA in breast cancer. Specifically, we evaluated serum IL2Rα levels in patients with breast cancer versus those with benign breast disease, assessed its diagnostic and prognostic utility, identified its cellular sources within tumor tissues, and explored its associations with immune cell infiltration. Additionally, we compared the expression patterns and clinical correlations of IL2RA and IL‐2 to elucidate their functional roles in breast cancer pathophysiology. These findings may contribute to the identification of novel diagnostic biomarkers and therapeutic targets for breast cancer.

## 2. Materials and Methods

### 2.1. Participants

This study was approved by the institutional review board of Shanghai General Hospital affiliated to Shanghai Jiao Tong University. A total of 252 consecutive patients who received breast surgery in Department of General Surgery in Shanghai General Hospital were retrospectively analyzed. Patients should be with a definite pathological diagnosis of breast cancer or benign breast disease. Complete clinical data and preoperative serum cytokine detection results available were required. Exclusion criteria include patients with other malignant tumors, severe autoimmune diseases, or infectious diseases; patients who received preoperative chemotherapy, radiotherapy, or targeted therapy; and patients with incomplete clinical or laboratory data. All tested parameters including serum cytokines and complete blood count were routinely analyzed in our center since 2020. The requirement for written informed consent was waived due to the retrospective nature of this study. Clinical data, including basic demographic information, underlying diagnosis, and histopathologic information, were collected from the participants and their medical records. All procedures involving human participants were in accordance with the ethical standards of national research committee and with the 1964 Helsinki declaration and its later amendments or comparable ethical standards.

### 2.2. Data Collection

The measurement of cytokines was performed in the Department of Clinical Laboratory, Shanghai General Hospital, using peripheral blood samples collected by trained nurses before surgery. Cytokines were tested by electrochemiluminescence immunoassay using Cobas E601 analyzers (Hoffman‐La Roche Ltd, Basel, Switzerland). The detection ranges for serum IL2Rα were 100–10,000 U/mL. Quality control was performed using low‐ and high‐level commercial control materials according to the manufacturer’s instructions, and all intra‐assay and inter‐assay coefficients of variation (CV) were < 5%.

TIMER database (version 2.0) (https://cistrome.shinyapps.io/timer/), incorporating 10009 samples with 23 cancer types based on TCGA, was used to compare IL2RA and IL2 expression in various cancers between cancer tissues and their matched normal tissues and to analyze the infiltration levels of immune cells including B cells, CD4+ T cells, CD8+ T cells, neutrophils, macrophages, and dendritic cells (DCs) based on gene expression profiles [15]. Kaplan–Meier plotter database (version 2024) (http://kmplot.com/analysis/) was used to analyze association between IL2RA and IL2 mRNA expression with relapse free survival (RFS) in breast [16]. Breast Cancer Gene‐Expression Miner v4.1 (bcGenExMiner v4.1) was utilized to evaluate the correlation between gene expression and clinicopathological parameters in breast cancer [17].

### 2.3. Statistical Analysis

Continuous data are presented as the means ± standard error of the mean. Independent samples *t*‐test was used for comparison between two groups. One‐way ANOVA was applied for comparisons among multiple clinicopathological subgroups. Survival analysis of serum IL2Rα was performed based on breast cancer‐specific survival (BCSF). All statistical analyses were performed using IBM SPSS Statistics 23.0 (IBM Corporation, Armonk, NY, USA) and GraphPad Prism 7.0 (GraphPad Software, CA, USA). *p* values of less than 0.05 were considered as statistically significant.

## 3. Results

### 3.1. Serum Levels of Cytokines in Women With and Without Breast Cancer

Firstly, we investigated cytokines serum levels in women with or without breast cancer. We recruited consecutive 99 breast cancer patients and 153 patients with benign breast disease in this study. Of the 99 breast cancer patients, 79 (79.8%) were histologically identified as invasive ductal carcinomas, 8 (8.1%) as ductal carcinomas in situ, 3 (3.0%) as invasive lobular carcinomas, 2 (2.0%) as Paget’s disease of breast, 2 (2.0%) as invasive papillary carcinoma, 2 (2.0%) as micropapillary carcinoma, 2 (2.0%) as malignant phyllodes tumor, and 1 (1.0%) as mucinous carcinoma. Out of 153 benign breast disease patients, 70 (45.8%) were histologically identified as fibroadenoma, 31 (20.3%) as fibrocystic mastopathy, 14 (9.2%) as duct ectasia, 14 (9.2%) as papilloma, 6 (3.9%) as granulomatous mastitis, 3 (2.0%) as sarcoidosis, 3 (2.0%) as cysts, 3 (2.0%) as focal fibrosis, 3 (2.0%) as phyllodes tumor, 2 (1.3%) as hamartomas, 2 (1.3%) as accessory breast, 1 (0.7%) as fat necrosis, and 1 (0.7%) as lipomas.

**FIGURE 1:**
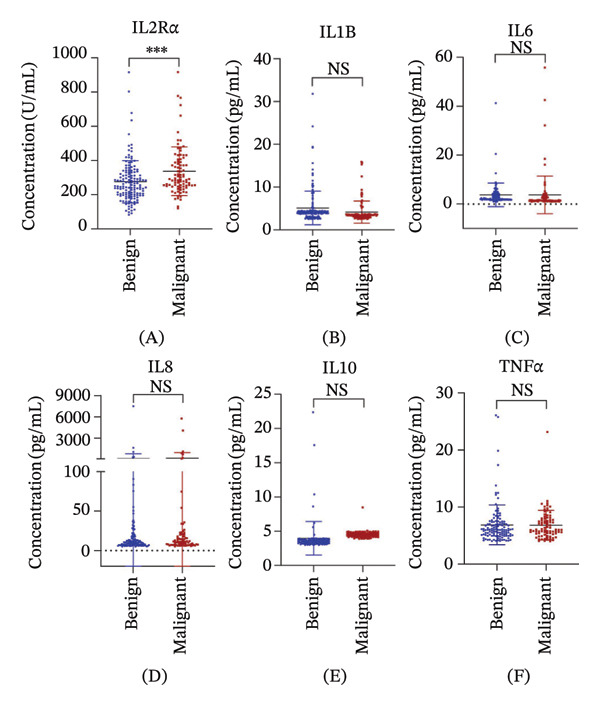
Serum cytokine levels in patients with breast cancer and benign breast disease. Comparison of serum concentrations of cytokines IL2Rα (1A), IL1β (1B), IL6 (1C), IL8 (1D), IL10 (1E), and TNFα (1F) between 99 breast cancer patients and 153 patients with benign breast disease. Only IL2Rα showed a significant elevation in breast cancer patients (benign: 275.8 U/mL vs. malignant: 336.9 U/mL, *p* < 0.001), while IL1β, IL6, IL8, IL10, and TNFα exhibited no statistically significant differences between the two groups.

Serum levels of cytokines in women with or without breast cancer are shown in Figure 1. The average serum concentration of IL1β (benign 5.1 pg/mL vs malignant 4.1 pg/mL, *p* = 0.152), IL6 (benign 3.7 pg/mL vs malignant 3.7 pg/mL, *p* = 0.982), IL8 (benign 105.5 pg/mL vs malignant 174.5 pg/mL, *p* = 0.531), IL10 (benign 4.0 pg/mL vs malignant 4.5 pg/mL, *p* = 0.152), and TNFα (benign 6.9 pg/mL vs malignant 6.8 pg/mL, *p* = 0.909) had no significant difference between women with and without breast cancer, while the average serum concentration of IL2Rα was significantly higher in breast cancer patients (benign 275.8 U/mL vs malignant 336.9 U/mL, *p* < 0.001).

### 3.2. Predictive and Prognostic Value of Serum IL2Rα in Breast Cancer

**FIGURE 2:**
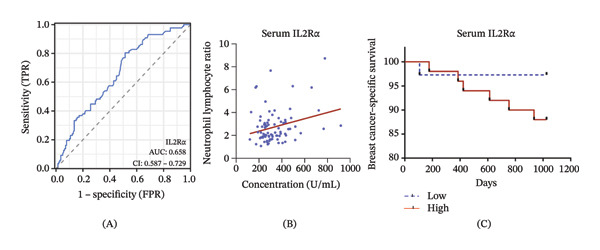
Diagnostic and prognostic value of serum IL2Rα in breast cancer. (A) Receiver operating characteristic (ROC) curve analysis of serum IL2Rα for distinguishing between benign and malignant breast diseases. The optimal cut‐off value was determined as 240.5 U/mL (Youden index = 0.258), with an area under the curve (AUC) of 0.650 (*p* < 0.001). (B) Correlation analysis between serum IL2Rα levels and neutrophil–lymphocyte ratio (NLR) in breast cancer patients, showing a significant positive correlation (*p* = 0.012). (C) Survival curve illustrating the association between serum IL2Rα levels and survival outcomes in breast cancer patients. Higher IL2Rα levels trended toward poorer survival (HR = 0.309, 95% CI: 0.069–1.381).

Given the elevated serum concentration of IL2Rα observed in breast cancer patients, this study further evaluated its predictive and prognostic performance in breast cancer. Receiver operating characteristic (ROC) curve analysis was first performed to assess the diagnostic utility of serum IL2Rα (Figure 2A). The optimal cut‐off value for IL2Rα was determined as 240.5 U/mL, which yielded the highest Youden index of 0.258. Based on the area under the curve (AUC) value of 0.650 (*p* < 0.001), serum IL2Rα exhibited moderate diagnostic power in distinguishing between benign and malignant breast diseases.

The neutrophil–lymphocyte ratio (NLR), a well‐recognized inflammatory biomarker, has been widely reported to correlate with tumor pathological characteristics. We therefore investigated the association between serum IL2Rα levels and NLR in breast cancer patients, revealing a significant positive correlation (*p* = 0.012) (Figure 2B). This finding suggests a potential link between IL2Rα and systemic inflammatory responses in breast cancer.

In terms of prognostic significance, a numerical trend indicated that higher serum IL2Rα levels were associated with poorer survival outcomes (*p* = 0.124, HR = 0.309, 95% CI: 0.069–1.381) (Figure 2C), though the wide confidence interval suggests the need for larger cohort validation. Collectively, these results indicate that serum IL2Rα not only serves as a potential diagnostic indicator for differentiating breast disease status but also may be linked to inflammatory processes and survival outcomes in breast cancer, highlighting its multifaceted clinical relevance in this malignancy.

### 3.3. Cellular Source of IL2Rα and Its Association With Immune Infiltration in Breast Cancer Tissues

**FIGURE 3:**
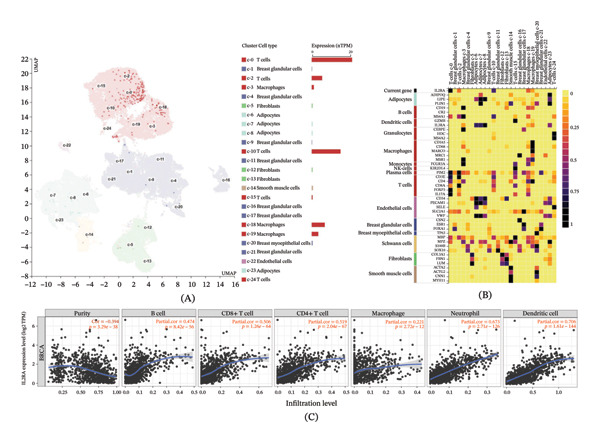
Cellular source of IL2Rɑ and its correlation with immune cell infiltration in breast cancer tissues: (A) scatter plot and (B) heatmap of single‐cell sequencing analysis indicating the cellular origin of IL2Rα in breast cancer tissues, with predominant expression in T cells and a smaller proportion in macrophages. (C) Scatter plots showing the correlation between IL2RA expression and infiltration of various immune cell types, including B cells (*r* = 0.474, *p* = 8.42*e* − 56), CD8+ T cells (*r* = 0.506, *p* = 1.26*e* − 64), CD4+ T cells (*r* = 0.519, *p* = 2.04*e* − 67), macrophages (*r* = 0.221, *p* = 2.72*e* − 12), neutrophils (*r* = 0.673, *p* = 2.71*e* − 126), and dendritic cells (DCs) (*r* = 0.706, *p* = 1.61*e* − 144).

To further clarify the cellular source of IL2Rα within breast cancer tissues, single‐cell sequencing data from breast cancer samples were analyzed. The results demonstrated that IL2RA is predominantly expressed by T cells, with a smaller proportion derived from macrophages, in breast cancer tissues (Figure 3A,B), which aligns with the earlier observed correlation between serum IL2Rα levels and systemic inflammatory markers, providing a potential cellular basis for the aforementioned findings.

Subsequently, we explored the relationship between IL2RA expression and immune cell infiltration in breast cancer. Comprehensive analyses revealed that high IL2RA expression was significantly associated with increased infiltration of multiple immune cell populations (Figure 3). Specifically, strong positive correlations were observed with B cells (*r* = 0.474, *p* = 8.42*e* − 56), CD8+ T cells (*r* = 0.506, *p* = 1.26*e* − 64), CD4+ T cells (*r* = 0.519, *p* = 2.04*e* − 67), and macrophages (*r* = 0.221, *p* = 2.72*e* − 2). Notably, the strongest correlations were observed with neutrophils (*r* = 0.673, *p* = 2.71*e* − 126) and DCs (*r* = 0.706, *p* = 1.61*e* − 144) (Figure 3C).

These findings collectively suggest that T cells as the primary sources of IL2Rα in breast cancer tissues may recruit neutrophils and DCs through IL2RA‐mediated signaling. This immune cell recruitment cascade could potentially modulate the tumor immune microenvironment and thereby promote breast cancer progression, establishing a functional link between IL2RA expression, immune infiltration patterns, and the pathological processes of breast cancer.

### 3.4. Expression Patterns and Clinical Associations of IL2RA and Its Ligand IL‐2 in Breast Cancer

**FIGURE 4:**
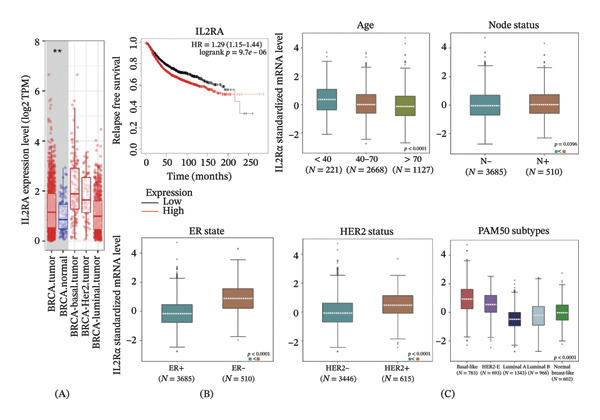
Expression pattern and clinical associations of IL2RA in breast cancer: (A) RNA‐seq analysis showing IL2RA expression levels in breast cancer tissues versus normal tissues, with significantly higher expression in tumor tissues. (B) Survival curve demonstrating that high IL2RA expression in tumor tissues is associated with poor prognosis in breast cancer patients (HR = 1.29, *p* = 9.7*e* − 06). (C) Association of IL2RA expression with clinicopathological features, including younger age at onset, lymph node metastasis, estrogen receptor (ER) negativity, HER2 positivity, and enrichment in basal‐like and HER2‐positive breast cancer subtypes.

Analysis of RNA‐seq data was performed to investigate the expression level of IL2RA and its relationship with clinicopathological characteristics. The results revealed that IL2RA is highly expressed in breast cancer tissues while showing low expression in normal tissues (Figure 4A). High expression of IL2RA in tumor tissues was associated with poor prognosis in breast cancer patients (*p* = 9.7*e* − 06, HR = 1.29, 95% CI: 1.15–1.44) (Figure 4B). Further analysis demonstrated that high IL2RA expression correlated with younger age at onset, lymph node metastasis, estrogen receptor (ER) negativity, and HER2 positivity. Thus, high IL2RA expression was more frequently observed in basal‐like and HER2‐positive breast cancer subtypes (Figure 4A,C).

**FIGURE 5:**
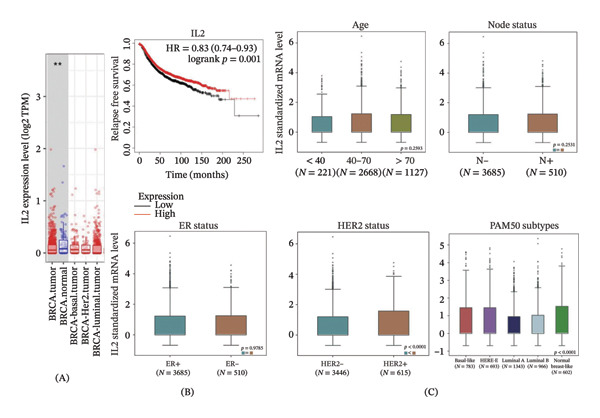
Expression pattern and clinical associations of IL‐2 in breast cancer: (A) RNA‐seq analysis showing IL‐2 expression levels in breast cancer tissues versus normal tissues, with higher expression in normal tissues. (B) Survival curve indicating that high IL‐2 expression is associated with better prognosis in breast cancer patients (HR = 0.83, *p* = 0.001). (C) Correlation of IL‐2 expression with clinicopathological features, showing no significant association with age, lymph node status, or ER status but slightly higher expression in HER2‐positive breast cancers.

Interestingly, its ligand, IL‐2, acts as an opposing marker in breast cancer. Analysis showed that IL‐2 expression was higher in normal breast tissues than in tumor tissues (Figure 5A), and high IL‐2 expression was associated with a better prognosis in breast cancer patients (*p* = 0.001, HR = 0.83, 95% CI: 0.74–0.93) (Figure 5B). IL‐2 expression levels did not correlate with age, lymph node status, or ER status and only exhibited slightly higher expression in HER2‐positive breast cancers (Figure 5C).

These findings suggest that IL2RA and IL‐2 may function as functionally opposing regulators in breast cancer, and their distinct expression patterns and clinical associations collectively highlight the potential significance of the IL‐2/IL2RA signaling axis in breast cancer pathophysiology.

## 4. Discussion

This study comprehensively investigated the role of IL2RA in breast cancer, encompassing serum expression patterns, diagnostic and prognostic value, cellular origins, immune microenvironment associations, and functional interactions with its ligand IL‐2. These observations support that IL2Rα modulates breast cancer progression by regulating immune cell infiltration and inflammatory response, revealing its key role in the tumor immune microenvironment.

### 4.1. Clinical Significance of Serum IL2Rα

A key observation that serum IL2Rα levels was significantly elevated in breast cancer patients is consistent with prior reports of elevated soluble IL‐2R in familial breast cancer [12] and extends these findings by demonstrating its potential as a diagnostic marker for differentiating malignant from benign breast conditions. The moderate diagnostic power of IL2Rα, with an optimal cut‐off of 240.5 U/mL (Youden index = 0.258), suggests that it may serve as a complement existing biomarkers in clinical practice, particularly in settings where invasive diagnostics are challenging. Given its moderate diagnostic performance (AUC = 0.650), serum IL2Rα is more suitably used as a complementary auxiliary biomarker in combination with existing clinical indicators, rather than an independent standalone diagnostic marker.

Notably, serum IL2Rα correlated positively with NLR (*p* = 0.012), a well‐established inflammatory marker linked to tumor progression. This association supports a link between IL2Rα and systemic inflammation, consistent with its role in T cell activation [11]. While higher serum IL2Rα trended toward poorer survival (HR = 0.309), the wide confidence interval (0.069–1.381) indicates the need for validation in larger cohorts, underscoring the importance of prospective studies to confirm its prognostic utility.

### 4.2. Cellular Origins and Immune Microenvironment Interactions

Single‐cell sequencing revealed that IL2RA is predominantly expressed by T cells (with a smaller contribution from macrophages) in breast cancer tissues. This cellular distribution provides a mechanistic basis for the observed correlation between serum IL2Rα and systemic inflammation, as activated T cells and macrophages are key producers of inflammatory mediators. Moreover, high IL2RA expression strongly correlated with infiltration of multiple immune cell types, including B cells, CD4+ and CD8+ T cells, macrophages, neutrophils, and DCs. The particularly robust correlations with neutrophils (*r* = 0.673) and DCs (*r* = 0.706) suggest that T cell–derived IL2Rα may orchestrate immune cell recruitment, shaping the tumor immune microenvironment.

This immune recruitment cascade could promote breast cancer progression by modulating inflammation or immune suppression. For instance, neutrophils are known to contribute to tumor angiogenesis and metastasis, while DCs can either enhance antitumor immunity or facilitate immune evasion depending on their activation state [18, 19]. Thus, IL2Rα may act as a central regulator of immune infiltration, influencing tumor behavior through complex interactions with the microenvironment.

### 4.3. Opposing Roles of IL2RA and IL‐2

A striking finding was the opposing expression patterns and clinical associations of IL2RA and its ligand IL‐2. This functional antagonism aligns with the dual roles of the IL‐2/IL‐2R axis in immunity: IL‐2 can promote antitumor responses through T cell activation but may also support regulatory T cell–mediated immune suppression [14]. The opposing expression and prognostic patterns between IL2Rα and IL‐2 indicate a functional decoupling of the IL‐2/IL‐2R axis in breast cancer, which may skew the immune milieu toward tumor promotion.

IL2RA’s association with aggressive clinicopathological features—younger age, lymph node metastasis, ER negativity, HER2 positivity, and basal‐like/HER2‐positive subtypes—further supports its role in promoting aggressive disease. In contrast, IL‐2 showed minimal correlation with clinical variables, except for slightly higher expression in HER2‐positive tumors, hinting at subtype‐specific roles that warrant further investigation.

### 4.4. Limitations and Future Directions

This study has several limitations. First, its retrospective, single‐center design may introduce selection bias, and the modest sample size limits the robustness of survival analyses. Although this was a single‐center retrospective study, the study cohort included a broad spectrum of breast cancer subtypes and benign breast diseases, which may ensure a certain level of sample representativeness. However, multicenter prospective studies are still needed to further validate our findings. Second, the lack of longitudinal data precludes assessing how IL2Rα levels change with treatment (e.g., surgery or chemotherapy) or their utility in monitoring disease progression [20, 21]. Third, mechanistic studies are needed to confirm whether IL2Rα directly mediates immune cell recruitment and to determine the downstream signaling pathways involved.

Future research should validate these findings in multicenter prospective cohorts, explore IL2RA’s utility in combination with other biomarkers, and investigate its potential as a therapeutic target—for example, through agents that block IL2Rα‐mediated immune recruitment. Additionally, studies on the IL‐2/IL2RA axis in specific breast cancer subtypes could reveal subtype‐specific therapeutic vulnerabilities.

## 5. Conclusion

Our findings establish IL2RA as a clinically relevant biomarker in breast cancer, with potential diagnostic and prognostic value. Its association with systemic inflammation, immune cell infiltration, and aggressive disease features, combined with its opposing relationship with IL‐2, highlights the IL‐2/IL2RA axis as a critical regulator of breast cancer pathophysiology. These insights may inform the development of novel complementary diagnostic tools and immunotherapeutic strategies targeting the tumor immune microenvironment. In clinical practice, serum IL2Rɑ may be used as a complementary biomarker for the auxiliary diagnosis of breast cancer and for monitoring systemic inflammation and immune status, which could help improve risk stratification and individualized management of breast cancer patients.

## Funding

This study was funded by Beijing Medical Foundation, MDK2023‐1009; National Natural Science Foundation of China, 82403399.

## Conflicts of Interest

The authors declare no conflicts of interest.

## Data Availability

The data that support the findings of this study are available from the corresponding author upon reasonable request.
